# Trends in research on indoor radon exposure and lung cancer in South Korea

**DOI:** 10.1186/s40557-016-0100-9

**Published:** 2016-03-08

**Authors:** Dae Ryong Kang, Dongmug Kang, Kyoung-Bok Min, Changsoo Kim, Sung-Soo Oh, Sang-Baek Koh

**Affiliations:** Department of Humanities and Social Medicine, Ajou University School of Medicine, Suwon, Korea; Department of Occupational and Environmental Medicine, Pusan National University, Yangsan Hospital, Yangsan, Korea; Department of Preventive Medicine, College of Medicine, Seoul National University, Seoul, Korea; Department of Preventive Medicine, Yonsei University College of Medicine, Seoul, Korea; Department of Occupational and Environmental Medicine, Wonju Severance Christian Hospital, Wonju College of Medicine, Yonsei University, Wonju, Korea; Department of Preventive Medicine and Institute of Occupational and Environmental Medicine, Wonju College of Medicine, Yonsei University, Wonju, Korea

## Editorial

Radon is recognized by the World Health Organization as a lung cancer-causing carcinogen in humans, and is the second most common cause of lung cancer after smoking [1]. A causal relationship between radon and lung cancer was first described in epidemiological studies on underground mine workers. Thereafter, case–control studies further demonstrated that exposure to low concentrations of radon carries a risk for lung cancer. Recently, pooling studies have set out to investigate lung cancer risk in relation to exposure to radon indoors.

In South Korea, a total of 47,869 men died from cancer in 2014, comprising 32.5 % of all male deaths that year. Among these Korean men, lung cancer had the highest mortality rate, compared to all other cancer types, and accounted for 12,785 deaths, corresponding to 26.7 % of all deaths from cancer. Meanwhile, in the same year, a total of 28,742 women died from cancer in South Korea, comprising 23.9 % of all deaths among women. As in men, lung cancer exhibited the highest mortality rate, accounting for 4655 deaths (16.2 % of all deaths from cancer) [2]. As the incidence of lung cancer has continued to increase, studies have been undertaken to outline the etiology thereof.

Histologically, lung cancer is primarily classified into small cell lung cancers (about 20 % of all lung cancers) and non-small cell lung cancers (80 %); non-small cell lung cancer is further subdivided into adenocarcinoma (40 %), squamous cell carcinoma (30 %), and large cell carcinoma (15 %). Small cell lung cancer is typically malignant, and in most cases, metastasizes to other organs before it is detected. Generally, smoking is considered to be the main cause of lung cancer: small cell lung cancers and squamous cell carcinoma are positively associated with smoking, whereas adenocarcinoma exhibits relatively lower correlation with smoking [2–4]. Meanwhile, however, 10–25 % of all cases of lung cancer are not directly related to smoking [5]. Reports suggest that passive smoking increases the risk of lung cancer by 35 % in men and 25 % in women [6]. Lung cancer has also been found to be related with radon exposure, exposure to potential lung carcinogens (asbestos, polycyclic hydrocarbons, silica, heavy metals, etc.), oil vapor when cooking, coal combustion, hormonal factors, and air pollution [7].

Despite awareness of radon’s association with lung cancer, Koreans are less aware of the potential risks of indoor radon exposure. This paper aimed to review trends in studies on indoor radon exposure and lung cancer in South Korea, to suggest the need to establish reference levels for indoor radon levels specific to South Korea, and to highlight the necessity of continuous indoor radon exposure-related research.

In this article, we outline eight papers published on indoor radon exposure and lung cancer with respect to Korea, covering topics related to epidemiological studies, mathematical-statistical modeling, environmental burden of disease, genetics biomarkers, and radon measurement methods.

The following summarizes our findings from reviewing research on indoor radon exposure:

- Ecological studies on indoor radon exposure and lung cancer in areas with high radon concentrations and where lung cancer incidence is high have emphasized a need for cohort and case–control studies. Furthermore, the following statistical methods for analyzing geographic information systems data were found to be employed: distance calculations, spatial aggregation, clustering, spatial smoothing and interpolation, and spatial regression [8].
- Studies in Germany, France, and Switzerland reported indoor radon concentrations similar to those in South Korea. Accordingly, the incidence of lung cancer in South Korea is likely to be affected by indoor radon exposure. One study has reported that about 5 % of all cases of lung cancer in South Korea might be attributable to radon [9]. Another study in Korea analyzed lung cancer-related mortality by region from 2000–2002 in relation to mean radon concentrations for 16 regions from 1989–2009 through a risk modeling method, using exposure, age, and duration, as well as exposure, age, and concentration. Their results were similar to those in other international studies. Most international studies have been designed as case–control studies, although cohort studies on the association between indoor radon and lung cancer have been conducted. These cohort studies have investigated the contribution of radon to lung cancer incidence using modeling methods. Nevertheless, there has been no such cohort study in South Korea so far.
- Upon reviewing whether residential radon exposure is associated with an increased risk for developing lung cancer and is regulated by genetic polymorphism in lung cancer in never smokers (LCINS) [10], we suggest that next-generation sequencing (NGS) may enable the detection of lung cancer-related genetic mutations in LCINS patients exposed to radon. Optimizing exom sequencing for use in NGS platforms is needed to analyze LCINS-related genes in normal and lung cancer tissues.
- To outline factors affecting indoor radon levels, mathematical models should be designed to apply time-dependent functions for indoor radon concentrations, and stochastic models await development. Applying the dynamic model of Radon Generation, Entry, and Accumulation indoors (RAGENA), which describes all known sources of radon, including soil, building materials, and water, it may be possible to obtain a mathematical model with which to estimate indoor radon concentrations [11].
- In reviewing the environmental burden of disease (EBD) attributable to residential radon exposure, we found that smoking status modifies the risk of lung cancer associated with radon; however, EBD among never smokers has not been explored [12]. Furthermore, no study has attempted to estimate the EBD of residential radon among never smokers in South Korea. As well, although residential radon exposure levels vary depending on the age of a building, studies have failed to account for the age of buildings in their assessments. In conclusion, studies that reflect disability weights and the age of buildings in Korea are required to obtain more precise estimates of the EBD of radon exposure indoors.
- Investigating and comparing methods of radon measurement, we noted that USA and Canada employ similar methods thereof in primary and secondary testing. In the UK, only long-term radon measurements are taken with seasonal adjustments [13]. In South Korea, both long-term and short-term measurements are taken in only primary testing. Standardization and improvement of domestic radon measurement methods still need to be studied.

Radon is invisible and a threat to human health. However, awareness about radon and its effects on humans is remains low among the general public. Hence, further studies to obtain accurate information with which to increase awareness about radon are needed. Currently, for Korea, data have been acquired from investigations of radon levels throughout the country, and though they could potentially be useful, indoor radon levels have not been measured in detail. Thus, if such data become available and the relationships between indoor radon exposure and lung cancer are effectively evaluated, it should be possible to set reference levels for indoor radon concentrations and to establish relevant mitigation policies and environmental health policies in South Korea. We suspect that such measures would help reduce the incidence of lung cancer and raise the quality of life of people throughout the nation.
