# Erratum to: Trends in research on indoor radon exposure and lung cancer in South Korea

**DOI:** 10.1186/s40557-016-0156-6

**Published:** 2016-12-28

**Authors:** Dae Ryong Kang, Dongmug Kang, Kyoung-Bok Min, Changsoo Kim, Sung-Soo Oh, Sang-Baek Koh

**Affiliations:** 1 Department of Humanities and Social Medicine, Ajou University School of Medicine, Suwon, Korea; 2 Department of Occupational and Environmental Medicine, Pusan National University, Yangsan Hospital, Yangsan, Korea; 3 Department of Preventive Medicine, College of Medicine, Seoul National University, Seoul, Korea; 4 Department of Preventive Medicine, Yonsei University College of Medicine, Seoul, Korea; 5 Department of Occupational and Environmental Medicine, Wonju Severance Christian Hospital, Wonju College of Medicine, Yonsei University, Wonju, Korea; 6 Department of Preventive Medicine and Institute of Occupational and Environmental Medicine, Wonju College of Medicine, Yonsei University, Wonju, Korea

## Erratum

In this version of this article that was originally published [1] the Acknowledgements section was missing and is included correctly below:

Acknowledgements

This subject is supported by Korea Ministry of Environment (MOE) as “the Environmental Health Action Program” (Grant Number 2015001350002).

The publisher apologises for these errors.
